# Supplementary material for: miR-7 Regulates GLP-1-Mediated Insulin Release by Targeting β-Arrestin 1
Source: Cells. 2020 Jul 6;9(7):1621. doi: 10.3390/cells9071621 (PMC7407368; doi:10.3390/cells9071621)
Supplement: Supplementary file 1 [file cells-09-01621-s001.pdf]

Supplementary Material

miR-7 Regulates GLP-1-Mediated Insulin Release by Targeting β-Arrestin 1

Alessandro Matarese [1,2,†], Jessica Gambardella [1,3,†], Angela Lombardi [1,4,†], Xujun Wang [1,5] and Gaetano Santulli [1,3,5*]

1 Department of Medicine, Fleischer Institute for Diabetes and Metabolism (FIDAM), Einstein-Mount Sinai Diabetes Research Center (ES-DRC), Albert Einstein College of Medicine, New York, NY 10461, USA; jessica.gambardella@einsteinmed.org (J.G.);
2 AORN "Antonio Cardarelli", 80100 Naples, Italy; alessandromatarese@yahoo.it (A. M.);
3 Department of Advanced Biomedical Science, "Federico II" University, and International Translational Research and Medical Education Consortium (ITME), 80131 Naples, Italy;
4 Department of Microbiology and Immunology, Albert Einstein College of Medicine, New York, NY 10461, USA; angela.lombardi@einsteinmed.org (A.L.);
5 Department of Molecular Pharmacology, Albert Einstein College of Medicine, New York, NY 10461, USA; xujun.wang@einsteinmed.org (X.W.)
* Correspondence: gaetano.santulli@einsteinmed.org; Tel.: 718-430-3370
† These authors contributed equally to this work.

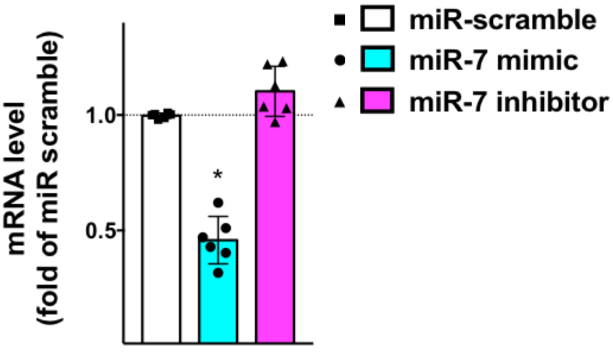

Supplementary Figure S1. βARR1 expression in pancreatic β cells is reduced by miR-7. βARR1 mRNA levels were measured in INS-1 β cells transfected with miR-7 mimic, inhibitor, or scramble (negative control) for 48 hours. Means ± S.E.M. are shown alongside actual values; *:$p<0.05$ *vs* miR-scramble.

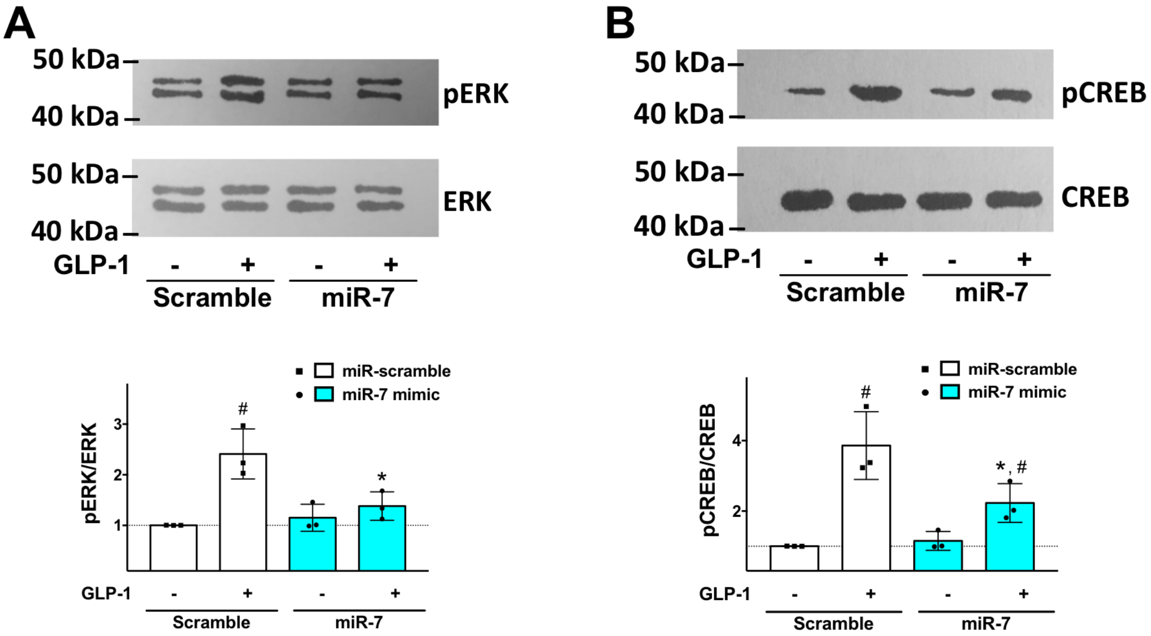

Supplementary Figure S2. GLP-1-induced phosphorylation of ERK and CREB is significantly attenuated by miR-7. Forty-eight hours after transfection with miR-7 mimic or miR-scramble (negative control), INS-1 β cells were treated with GLP-1 (100 nM) for 10′ and the activation of ERK (A) and CREB (B) was assessed in cell lysates by immunoblot. Representative immunoblots from three independent experiments are shown. Means ± S.E.M. are shown alongside actual values; *:p<0.05 *vs* scramble; #:p<0.05 *vs* no GLP-1 (vehicle).

Supplementary Table S1 – Sequences of oligonucleotide primers and product sizes

	Primer	Sequence (5′-3′)	Amplicon (bp)
**βARR1**	*Forward*	ACG CCA AGA AAG GAG TCT CA	81
	Reverse	ATT TAG CCA AGC ACC ACC AC	
**GAPDH**	*Forward*	TGC CAC TCA GAA GAC TGT GG	85
	Reverse	GGA TGC AGG GAT GAT GTT CT	

βARR1: β Arrestin 1;

GAPDH: glyceraldehyde 3-phosphate dehydrogenase.
